# The role of exosomes in tumour immunity under radiotherapy: eliciting abscopal effects?

**DOI:** 10.1186/s40364-021-00277-w

**Published:** 2021-03-31

**Authors:** Tianwen Yin, Huixian Xin, Jinming Yu, Feifei Teng

**Affiliations:** 1 Department of Radiation Oncology, Shandong Cancer Hospital and Institute, Shandong First Medical University and Shandong Academy of Medical Sciences, Jinan, 250117 China; 2 Department of Radiation Oncology, Qilu Hospital of Shandong University, Jinan, 250012 China

## Abstract

As a curative treatment of localized tumours or as palliative control, radiotherapy (RT) has long been known to kill tumour cells and trigger the release of proinflammatory factors and immune cells to elicit an immunological response to cancer. As a crucial part of the tumour microenvironment (TME), exosomes, which are double-layered nanometre-sized vesicles, can convey molecules, present antigens, and mediate cell signalling to regulate tumour immunity via their contents. Different contents result in different effects of exosomes. The abscopal effect is a systemic antitumour effect that occurs outside of the irradiated field and is associated with tumour regression. This effect is mediated through the immune system, mainly via cell-mediated immunity, and results from a combination of inflammatory cytokine cascades and immune effector cell activation. Although the abscopal effect has been observed in various malignancies for many years, it is still a rarely identified clinical event. Researchers have indicated that exosomes can potentiate abscopal effects to enhance the effects of radiation, but the specific mechanisms are still unclear. In addition, radiation can affect exosome release and composition, and irradiated cells release exosomes with specific contents that change the cellular immune status. Hence, fully understanding how radiation affects tumour immunity and the interaction between specific exosomal contents and radiation may be a potential strategy to maximize the efficacy of cancer therapy. The optimal application of exosomes as novel immune stimulators is under active investigation and is described in this review.

## Background

As a crucial treatment for local tumours, radiotherapy (RT) has been used for more than a hundred years. RT not only affects the irradiated tumour but also elicits multiple immunomodulatory effects on both the tumour and tumour microenvironment (TME), committing the tumour to an immune-mediated response. RT elicits immunogenicity in irradiated cancer cells through the DNA damage response and can also shift the balance of the TME to an immunostimulatory state by promoting tumour antigen transfer, priming effector T cells, and increasing the number of natural killer (NK) cells. The upregulation of adhesion molecules and other cytokines can also contribute to immune activation during RT. Abscopal effects are systemic antitumour effects that occur as tumours outside the irradiated field regress, and these effects are rare and still not fully understood.

Recent studies have shown that exosomes play a crucial role in RT-associated immunity. Exosomes are implicated in the regulation of tumour cell metabolism, RT sensitivity and the transmission of RT resistance. Exosomes are nanometre-sized vesicles with multiple roles, such as particle conveyance, antigen presentation, and immunomodulation, that can function in an endocrine manner and even via direct contact. Exosomes can be found in almost all kinds of cells and are gradually becoming important bioactive molecule biomarkers, as they contain specific proteins, enzymes, and nucleic acids from the parent cells. To date, studies on exosomes have undergone much development since exosomes were first discovered thirty years ago. The understanding of exosomes extends from simple particle release to cellular communication and immunomodulation, especially in the context of tumours. In recent years, increasing studies have focused on the specific contents of exosomes, such as DNA, RNA, and proteins, which may be the functional components of exosomes. Studies have shown that stressful conditions affect the secretion, composition, abundance, and potential binding of exosomes to recipient cells [1]. Exosomes were considered immunosuppressive for a long time; however, accumulating evidence indicates that after irradiation (IR), the increased release and altered contents of exosomes from donor cells make these exosomes more oncogenic [2]. Exosomes affected by RT are largely immunostimulatory, which can lead to a systemic response, which explains the abscopal effect to some extent.

Although much progress has been made in elucidating exosome-mediated functions in vitro, there are still many problems to be faced in radiation oncology exosome research. Here, we summarize the roles of exosomes in tumour immunity during RT, which may explain the abscopal effect. The combination of exosomes and RT has wide application prospects in tumour diagnosis, prognosis, antitumour immunity enhancement, and radioresistance elimination and needs further exploration.

### The effect of radiotherapy on immunity

RT is a critical component of cancer treatment. For many patients with localized cancer, RT is essential for effective control and can result in a curative effect [3]. Many studies have focused on the mechanism of RT and have illustrated how the immune system reacts to IR in tumour patients. RT can exert direct cytotoxic effects on tumour cells and reprogramme the TME to exert an antitumour immune response. Radiation initiates immunogenic cell death and the production and release of a set of cytokines and chemokines into the TME, which leads to the infiltration of DCs, macrophages, cytotoxic T cells (CTLs), regulatory T cells (Tregs) and myeloid-derived suppressor cells (MDSCs) that can play suppressive roles [4, 5]. By killing tumour cells, triggering the release of proinflammatory factors, and upregulating tumour-infiltrating immune cells, RT can turn immunologically ‘cold’ tumours into ‘hot’ ones. Eventually, RT can regulate and change both malignant and benign components of the TME [6].

For several decades, the abscopal effect has been observed to be therapeutic in several cancers following RT of the localized primary tumour or a distant metastatic focus. Although the biological mechanism is still not fully understood, several studies have helped to elucidate that this effect may result from a combination of inflammatory cytokine cascades and immune effector cell activation induced by immunogenic cell death (ICD), which ultimately destroys unirradiated tumour cells [7, 8]. After IR of the tumour, injury to the tumour results in necrotic and apoptotic tumour cell debris, which may lead to the liberation of neoantigens, which are tumour-associated antigens (TAAs). These TAAs are captured by antigen-presenting cells (APCs) and then presented to CD8+ T cells, resulting in substantial increases in the number and diversity of TAAs that can stimulate robust tumour-specific immune responses in which specific CD8+ T cells recognize and attack both the primary tumour and metastatic disease [7, 9]. Irradiated tumour cells also trigger the production of cellular danger-associated molecular patterns (DAMPs) and release cytokines that enhance immune cell trafficking [10]. In summary, these factors support the priming of CD8+ T cells to eliminate tumour cells [11].

#### Enhancing immune responses

Accumulating evidence indicates that RT can augment both innate and adaptive immune responses against tumours, thereby decreasing immunosuppression and potentiating the responsiveness of tumours to RT [5, 12]. In a mouse fibrosarcoma model, Stone et al. found that the host immune status determined the efficacy of radiation-induced antitumour effects [13]. Studies of different tumour models show that T cells are required for radiation-induced tumour regression and that CD8+ T cells infiltrate contribute to the effects of RT [4]. The level of antigen presentation is upregulated after RT. Tumour-specific antigens can be recognized and processed by APCs and then elicit an antitumour response by specific T cells. Radiated tumour cells can undergo immunogenic death in which tumour cells die, effectively exposing tumour antigens and triggering an antitumour immune response [14]. Several studies have shown that RT can increase antigen presentation by cancer cells and modulate this process [6]. In a human melanoma cell line, after different doses of γ-radiation, a dose-dependent increase in major histocompatibility complex class I (MHC I) expression was observed. Additionally, Eric et al. showed that radiation could expand the intracellular peptide pool of irradiated cells in a dose-dependent manner and alter the MHC I-associated peptide profile. Furthermore, these radiation-induced increases in MHC I expression lead to increased sensitivity to antigen-specific CTL killing [15]. Radiation increases the number of unique T cell receptors (TCRs) and T cell clonality, and when this effect is combined with anti-programmed cell death 1 (PD-1) therapy, this increased diversity can extend to regions outside of the radiation field. Dendritic cells (DCs) are myeloid-derived cells that are profoundly affected by the TME and are altered by IR. Within the irradiated tumour fields, chemokines that attract antigen-specific T cells and DCs are released. The Lord lab and others have demonstrated that IR increases the levels of tumour-associated DCs, enhances the mobilization of these cells to draining lymph nodes, augments DC maturation, increases the cross-presentation of antigens and primes T cells. Radiation can increase the release of DAMPs from dying and stressed cells, such as calreticulin, high mobility group box 1 (HMGB1), and ATP, which are associated with ICD. These molecules bind with pattern recognition receptors and induce DC maturation, giving these cells the ability to present antigens effectively and modulate adaptive immunity in tumours [14]. By inducing cytosolic nucleic acid-sensing pathways (cGAS-STING-dependent pathways) to trigger type I interferon (IFN) signalling in DCs, RT can regulate the adaptive immune response [16].

NK cells are lymphocytes that are critical in host surveillance against tumours [17]. Engagement of the receptor natural-killer group 2 member D (NKG2D) activates NK cells, and studies have shown that in the presence of functional p53 and after radiation, specific NKG2D ligands are upregulated, which induce the potent activation of NK cells and provide strong co-stimulation to CD8+ T cells [18, 19].

In addition to mobilizing antitumour immunity, RT shifts the TME into a condition that contributes to effector T cell recruitment and function [20]. RT can increase chemokines that recruit effector T cells, which makes inflamed tumour tissue susceptible to T cell attack. In a mouse model of breast cancer, scientists found that IR markedly enhanced the secretion of C-X-C motif chemokine ligand 16 (CXCL-16), which binds to C-X-C motif chemokine receptor (CXCR6) on Th1 cells and activates CD8+ T cells. Thus, increased migration of activated CD8(+)CXCR6(+)T cells can be found in tumour tissue [21]. Previous studies have shown that RT can promote leukocyte-endothelial transmigration while enhancing cell surface intercellular adhesion molecule-1 (ICAM-1) expression on endothelial cells [22, 23]. After priming, tumour-specific T cells exit the lymph nodes and circulate in the body from the irradiated area to the nonirradiated area, thus playing a role in distant tumours and possibly contributing to abscopal responses [24, 25]. Moreover, a study showed that low doses of RT reprogrammed tumour-associated macrophages (TAMs), which are mostly the immunosuppressive M2 phenotype, to the M1 phenotype [26]. M1 macrophages can express a series of proinflammatory cytokines, including IL-12, IL-23, NO, and tumour necrosis factor (TNF)-α, and high levels of MHC-I and MHC-II. In this manner, low-dose RT can induce beneficial T cell conditions and have an antitumour effect [27]. Multiple studies have indicated that in many types of cancer patients and under different treatments, the presence of tumour-infiltrating lymphocytes, especially effector T cells, is associated with better outcomes [17, 28, 29]. The reason may be that RT induces the release of chemokines that subsequently enhance T cell infiltration and the priming of present and newly infiltrated T cells, which leads to positive immunological outcomes.

#### Activating immunosuppressive responses

RT can elicit an immunostimulatory effect by regulating antigen presentation, DC maturation, NK cell function, and CD8+ T cell infiltration. However, RT can also activate immunosuppressive signalling in normal cells. Tregs and TAMs are the main factors that play immunosuppressive roles in the TME. Both cell types can promote tumour growth and are associated with poor prognosis [30]. Tregs induce immunosuppression through cytotoxic T lymphocyte-associated protein 4 (CTLA4) signalling and the production of the cytokines transforming growth factor (TGF β) and interleukin (IL)-10, and adenosine [31, 32]. TAMs can create a supportive stroma for neoplastic cell expansion and invasion, further facilitating tumour proliferation, survival, and migration. In a head and neck squamous cell carcinoma (HNSCC) murine model, researchers found that RT could upregulate CCL2 production and induce the CCR2-dependent accumulation of Tregs and TNF α-producing monocytes and macrophages, thereby playing an immunosuppressive role [31].

The effect of radiation on immunity is complex due to the various changes in chemokines and cells in the TME, which is vital for cell-to-cell communication. In general, the low immunogenicity of tumour antigens, the prevalence of immunosuppressive cells such as MDSCs and Tregs, and immunosuppressive cytokines such as IL-10 and TGF β may work together to induce immunosuppression in the TME, and a specific threshold level of radiation can shift the balance towards immune activation. The immunogenicity of a given tumour is determined, the type and dose of radiation, fraction regimen, and other immune agents may cause antitumour immunity to vary. To induce an abscopal effect and make better use of radiation to stimulate antitumour immunity, more work needs to be done to fully understand the cell communication and changes induced by RT.

### The biogenesis, formation, and functions of exosomes

Exosomes are small extracellular vesicles with diameters < 150 nm that contain genetic materials, proteins, and lipids [33, 34]**.** Exosomes are generated via a dynamic process that involves double invagination of the plasma membrane and the formation of intracellular multivesicular bodies (MVBs) and intraluminal vesicles (ILVs) [35]. The plasma membrane first invaginates to form endosomes and subsequent ILVs. Late ILVs containing endosomes are referred to as MVBs, which are also called multivesicular endosomes [34]. During the formation of exosomes, their contents are directly sorted or sometimes stochastically acquired from cytoplasmic and membrane-bound contents [35]. The sorting mechanism occurs in both endosomal sorting complex required for transport (ESCRT)-dependent and ESCRT-independent manners, and these processes guide specific molecules into the exosomes derived from MVBs [36, 37]. Eventually, MVBs can fuse with the plasma membrane to release ILVs from the parent cell as exosomes or fuse with autophagosomes for degradation [35].

Exosomes are secreted by many kinds of cells, such as B lymphocytes, T cells, macrophages, DCs, and tumour cells [8], and can deliver their protein, lipid, and RNA cargoes to recipient cells via physiological and pathological processes, thereby functioning in cellular communication [38]**.** Paracrine and endocrine mechanisms are important means of intercellular communication mediated by exosomes [39]. Due to their universality, exosomes are abundant in the human body and can be found in biological fluids, including urine, tears, plasma, and breast milk, and in cell culture supernatants [40–42].

The distinct mechanisms associated with exosome secretion and uptake and exosomes from specific cell types result in a complex role of exosomes in intercellular communication [43, 44]**.** Moreover, since exosomes are generated from parent cells, their contents can reflect the status of the parent cell to some extent. Due to their specific lipid, DNA, proteins, and functional RNA contents, exosomes play crucial roles in cell-cell communication, antitumour immunity, tumour metastasis and tumour therapy resistance.

Because they are generated from parental cells, exosomes can reflect the status and character of the parental cells, and the idea that exosomes can be used as biomarkers for disease has attracted attention [38]**.** Existing stably in the TME, exosomes can be found in various body fluids, can be obtained in a non-invasive manner, and can reflect changes in the environment; thus, exosomes can be used as liquid biopsies for evaluating the therapeutic efficacy of disease treatments.

Despite the encouraging advances mentioned previously, many challenges and difficulties still exist in the clinical applications of exosomes. First, due to their low abundance, exosomes are difficult to detect accurately. Second, the complex and random conformation has made precise targeting of specific exosomes challenging, and isolating specific exosomes completely and efficiently still needs further exploration. With advanced technologies, improved experimental approaches, and further research, these problems will be solved, and exosomes will become widely used in the clinic.

### The immune effects of exosomes on tumours

Because of the role of exosomes in cell signalling and their specific contents, exosomes can take part in a series of processes in different tumour types [34]**.** Among them, the role of exosomes in immune responses has been widely documented, which may be clues to understanding tumour development. Exosomes are derived from different parental cells and may play an immunoregulatory role through tumour antigen presentation, the delivery of gene-expression contents to recipient cells, and the induction of signalling pathways via surface ligands [35].

#### Inducing antitumour immune responses

Many studies have indicated that exosomes can function in innate and adaptive immunity [35, 45]. By transferring antigens to APCs, tumour-derived exosomes (TEXs) and immune cell-derived exosomes (IEXs) can activate antigen-specific T cell responses, thereby enhancing antitumour responses [46]. To initiate T cell-mediated antitumour immune responses, DCs must take up and process tumour antigens which are then presented on MHC-I molecules [47]. TEXs originate from tumour cells and may contain many tumour-specific antigens, such as melan-A, gp 100, carcinoembryonic antigen (CEA), and mesothelin [47, 48]. It has been revealed that many TEXs isolated from malignant effusions can transfer these tumour antigens to DCs and induce specific CTL responses and antitumour immunity [48, 49]. When tumour cells are exposed to stress conditions, exosomes can also exhibit immune-activating functions. For example, heat-shocked tumour-derived exosomes express heat shock protein 70 (HSP70) on the surface, thereby improving the migration and cytotoxicity of NK cells and stimulating macrophages to secrete TNF [50, 51]. Another study showed that exosomes from heat-stressed CEA-positive tumour cells (CEA+/HS-Exos) contained CEA and increased levels of HSP70 and MHC-I molecules after HLA-A2.1/Kb transgenic mice were immunized with CEA+/HS-Exos, and a more efficient CEA-specific CTL response was observed [52].

Many IEXs have similar roles as their parent cells in activating antigen-specific T cell responses. Activated CD8+ T cells produce cytotoxic exosomes that directly target mesenchymal tumour stromal cells to prevent the invasion and metastasis of tumours [53]. In a tumour-bearing mouse model, peptide-pulsed DC-derived exosomes (DEXs) were used as a novel cell-free vaccine to eradicate tumours, as DEXs contain tumour antigens and present antigens to elicit potent antitumour immune responses [54]. Studies have demonstrated that DEXs can trigger potent CD8+ T cell-dependent antitumour responses by transferring exosomal molecules to DCs [47, 55]. In vivo, exosomes containing high levels of HSP60 and HSC70 are derived from mast cells, promote DC maturation and elicit specific immune responses in mice [56]. NK cells can play a central role in the immune response against cancer as innate lymphoid cells [57]. One study demonstrated that NK cells can release exosomes containing high levels of FAS-L and TNF-α to exert cytotoxic effects on melanoma cells [58]. These immunostimulatory effects support the utility of exosomes in therapy development and their potential role in coordinating with the immune reactions to cancer [35].

#### Inhibiting antitumour immune responses

Although we have mentioned that TEXs can be used as a source of tumour antigens to stimulate antitumour responses, additional evidence has shown that TEXs primarily suppress antigen-specific and nonspecific antitumour responses [59]. TEXs can play suppressive roles in T cells and NK cells, inducing a lethal effect on target tumour cells in an antitumour immune response. Research has shown that melanoma cells release FasL-bearing exosomes and trigger lymphoid cell apoptosis in a Fas-dependent manner [31]. Similarly, in patients with ovarian carcinoma, FasL-associated TEXs suppress CD3-ζ chain expression in T cells, inducing T cell apoptosis [60]. In a hypoxic tumour model, by upregulating miR-23a and transferring transforming growth factor-β (TGF-β) to NK cells, TEXs target CD107a and decrease the expression of NKG2D in NK cells, thereby inhibiting NK cell function [61].

TEXs induce monocytes to differentiate into MDSCs, which suppress T cell activation and function [62]. Membrane-bound programmed cell death ligand 1 (PD-L1) and the cytokine TGF-β are important molecules that are upregulated by TEXs to exert immunosuppressive effects [63–65]. Studies have shown that gastric cancer-derived exosomes induce monocytes to differentiate into PD-1+ TAMs, which can suppress antitumour responses by triggering PD-1/PD-L1 signalling [66]. Similarly, in a Lewis lung cancer model, TEXs inhibited myeloid precursor cell differentiation into DCs and restrain DC maturation by increasing the expression of PD-L1, which triggers inhibitor signals [67]. Through a TGF-β1-dependent mechanism, TEXs inhibited the maturation of macrophages and DCs in an experiment. In addition, by directly affecting Tregs, TEXs enhance Treg function and inhibit apoptosis. As a large part of the TME, TEXs play important roles in inhibiting antitumour immunity.

Despite originating from immune cells, IEXs may have characteristics that are different from the parent cells and play roles in tumour progression and metastasis. A study showed that exosomes expressing FasL derived from activated CD8+ T cells promote tumour progression and metastasis via the Fas/FasL, ERK and NF-κB pathways [68]. As observed in B cells, activated CD8+ T cells release CD19+ EVs, impairing CD8-specific T cell in tumours [69]. Mesenchymal stem cells (MSCs) also secrete immunosuppressive exosomes under various conditions. MSCs are a group of progenitor cells that are heterogeneous and exist in almost all tissues [70, 71]. It has been found that liver MSC-derived exosomes induce downstream TGFβ/Smad2/3 signalling in NK cells via TGFβ, thereby inhibiting the activation and reducing the cytotoxicity of NK cells [72]. MSC-derived exosomes also exert immunosuppressive effects on DCs primarily through the inhibition of DC maturation and activation [73].

#### Exosomal PD-L1

The abscopal effect is occasionally observed in the context of RT; however, this effect cannot be a therapeutic goal, as it is too rare and unpredictable to be seen reliably. The findings in some patients showing that the blockade of the main immune checkpoints, such as PD-1/L1 or CTLA-4, can contribute to immune-related tumour regression have reignited interest in cancer immunotherapy [74]. These findings have attracted attention regarding the use of radiation combined with immunotherapy to overcome cancer resistance. Immune checkpoint blockade therapy has become a new strategy in cancer treatment and has achieved much progress [75]. However, the low response rate of immune checkpoint blockade therapy is still its main restriction [76].

**Fig. 1:**
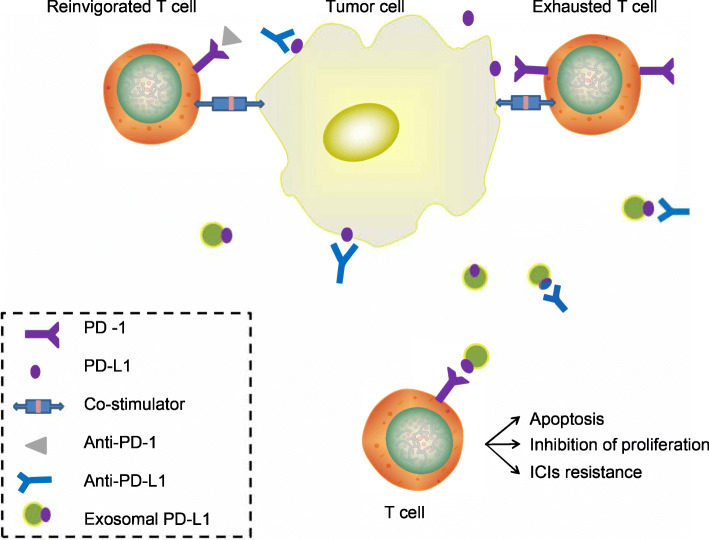
Exosomal PD-L1 contributes to resistance to ICI therapy. **a**. Tumour cells express surface PD-L1, which directly binds to PD-1 on T cells, eliciting an immune checkpoint response. After the immune checkpoint response, T cell activation is suppressed and leads dysfunction or apoptosis, which directly inhibits antitumour immunity. **b**. Immune checkpoint inhibitors (ICIs) can bind to immune checkpoints, such as PD-1/L1 and CTLA-4, and free exhausted T cells to rejuvenate antitumour immunity. **c**. Tumours upregulate the expression of PD-L1 to avoid activated T cell attack. Moreover, tumour cells release exosomal PD-L1, which has similar effects as tumour-derived PD-L1. Exosomal PD-L1 can bind to PD-1 on T cells and ICIs, thereby inhibiting T cell activation and proliferation and inducing T cell apoptosis and resistance to ICI therapy

Programmed death-ligand 1 (PD-L1), also known as CD274, is a cell surface membrane-bound ligand associated with many types of tumour cells. PD-1 is usually upregulated in many cancers [77]. PD-L1 binds to PD-1, which is also known as CD279, on T cells. By suppressing T cell activation, this pathway induces the immune evasion of tumour cells [63, 78]. In the TME, PD-L1 is expressed not only on tumour cells but also on DCs, macrophages, MDSCs, and many other cell types [79]. Among them, PD-L1 is present not only on the surface of tumour cells but also on the exosomal surface and within the vesicle structure [80, 81]. Exosomal PD-L1 can be found in many types of cancer, such as melanoma, breast cancer, glioblastoma, and lung cancer [80, 82–86]. Studies have shown that exosomal PD-L1 inhibits T cell activity, which is associated with its immunosuppressive effect on many types of cancer (Fig. 1).

A recent study demonstrated that in a prostate cancer model, tumour-derived exosomal PD-L1 could suppress T cell activation in the draining lymph node. Furthermore, after the removal of exosomal PD-L1, even in anti-PD-L1 antibody-resistant models, tumour growth could be inhibited [81]. Similarly, studies have shown that exosomes derived from lung cancer cells express PD-L1, and these exosomal PD-L1 molecules inhibit IFN-γ secretion and induce apoptosis in CD8 T cells to promote tumour growth and mediating immune escape [87]. Moreover, this immunosuppressive activity required the expression of both PD-L1 and MHC class I on exosomes [82].

Exosomes transfer functional PD-L1 in a dose-dependent manner to other cells that express no or little PD-L1 [88]. This may be one of the mechanisms facilitating tumour progression. A research team found that PD-L1-positive breast cancer cells transport PD-L1 to PD-L1-negative breast cancer cells and this transferred PD-L1 is then localized to the target cell surface [82]. Macrophages, DCs, and MDSCs that express PD-L1 can also be sources of exosomal PD-L1 and transfer this molecule to a number of cell types in vitro. However, the expression of exosomal PD-L1 on myeloid cells is critical in immunosuppression, and few reports have illustrated this effect.

The clinical value of exosomal PD-L1 is significant [89]. Because of its stable structure, exosomal PD-L1 is not easily degraded by proteolytic enzymes; moreover, the immunomodulatory effects of exosomal PD-L1 on the TME are robust. A series of studies showed that by combining the advantages of exosomes and PD-L1, tumour-derived exosomal PD-L1 has an durable immunosuppressive effect on tumours [82]. Therefore, we hypothesize that exosomal PD-L1 may be associated with a low response to immune checkpoint blockade therapy. This finding also reminds us of how to target exosomal PD-L1 optimally to augment the antitumour response and overcome resistance to PD1/PD-L1 blockade therapy. For example, we can inhibit exosome release to reduce the level of exosomal PD-L1 and combine this approach with anti-PD-L1 therapy to restore the specific T cell response. This strategy may have the potential to enhance the antitumour response in cancer patients.

Regardless of the immunostimulatory role, such as that of IEXs, or the immunosuppressive role of exosomes, such as that of exosomal PD-L1 in the tumour, these studies have illustrated the importance of exosomes in tumour development and progression, especially their potential use in cancer therapy. In short, these results provide us with a theoretical basis for exosomes as new treatment regimens. For example, targeting the exosome formation to obtain specific exosomes and developing methods for exogenous administration of specific exosomes to promote and enhance the immunostimulatory effect may be effective in controlling tumours.

### The interaction between radiotherapy and exosomes

As mediators of intercellular communication that transfer their protein, lipid, and nucleic acid cargo to recipient cells, exosomes have the ability to alter the molecular profiles of cells, signalling pathways, and even gene regulation [1]. Moreover, exosomes are a common part of the TME that can be easily affected by surrounding conditions. Scientists have demonstrated that different physiological and environmental conditions alter exosome composition [90]. Radiation can exert a direct effect on parental cells and induce stressful conditions, thereby exerting effects on the processes of exosome composition, secretion, transport, and function [52, 91].

Many studies have exhibited abscopal effects, which means that irradiated cells can exert ionizing radiation-induced effects on unirradiated cells by releasing exosomes, which then cause functional responses in recipient cells. Abscopal effects have been connected to some mechanisms involved in the immune system. Despite the promise of RT to induce abscopal effects to treat metastatic diseases, this phenomenon is rare because of the treatments and established immune tolerance mechanisms. Exosomes are crucial immune regulators, and it has been reported that exosomes are involved in different aspects of the radiation response, including transmitting abscopal effects and mediating radioresistance [92, 93].

#### Affecting the secretion and composition of exosomes

It has been observed in many types of normal and tumour cell lines that radiation affects the amount of exosome secretion, which then affects intercellular communication, which is exosome-based [92, 94, 95]. A recent study showed that in human keratinocytes and HacaT cell models, the number of exosomes increased with increasing doses of γ rays radiation (0.005, 0.05, and 0.5 Gy) [95]. Similarly, research has shown that treating a glioblastoma cell line (U87MG) with 2, 4, 6, and 8 Gy of X-ray radiation can cause time- and dose-dependent exosome release [96]. In another study, similar results were observed. L-Plastin is a vital factor that is responsible for the mitogenic and clonogenic activity of cells. It has been reported that radiation inhibits the release of exosomes containing L-Plastin, thereby achieving tumour control by radiation [2]. After RT, rising levels of circulating HSP72 and some proinflammatory cytokines in the serum of 13 prostate cancer patients was observed. The accompanying in vitro and mouse model studies showed that the released HSP72-containing exosomes played a vital role in this stimulating immune response [97]. These findings suggest that the upregulated release of HSP72-containing exosomes may be associated with enhanced RT efficacy and could be a potential therapeutic strategy for prostate cancer treatment.

Radiation can also alter the molecular compositions of exosomes. Previous studies have highlighted the ability of exosomes to transport tumour-suppressive and oncogenic molecules to recipient cells, thereby activating cellular downstream signalling pathways and impacting cell activities [98]. However, radiation can affect exosome composition to affect cellular signalling. Exosomes can reflect radiation-induced changes in cellular processes such as transient transcription suppression, translation and signalling induced by stress. After exposure to radiation (2 Gy), exosomes secreted from FaDu cells, which originate from HNSCC, upregulate the proteins involved in translation, chaperones, ubiquitination-related factors and proteasome components [93]. A recent study showed that following radiation, exosomes produced by mouse breast cancer cells transfer dsDNA to DCs, upregulate the costimulatory molecules on DCs, stimulate STING-dependent activation of IFN-I, and elicit tumour-specific CD8+ T antitumour responses in vivo [99]. Another study reported that radiation increased the abundance of exosomes released from glioblastoma cells and normal astrocytes [98]. In HeLa cervical carcinoma cell models, radiation can significantly affect exosome survivin release. Although the exosomal secretory rate was not influenced, the survivin content of isolated exosomes was higher than that of control group exosomes after proton IR (3 Gy) at a sublethal dose. This result suggests that RT-induced upregulation of exosomal survivin release may be associated with radioresistance and cancer recurrence and could be used as a potential therapeutic strategy in cervical cancer treatment [2].

Radiation influences exosome composition not only by altering immune status but also by changing cell migration. In glioblastoma cells, by elevating a group of molecules associated with cell migration signalling pathways, such as connective tissue growth factor (CTGF) mRNA and insulin-like growth factor binding protein 2 (IGFBP2), radiation enhanced the migration of recipient cells [93]. Similarly, one study showed that radiation regulated exosomal proteins to increase AKT signalling, thereby increasing phospho-mTOR, phospho-rpS6, and matrix metalloproteases (MMP)2/9 protease activity, ultimately promoting recipient cell movement and metastasis [100, 101].

#### Exosome-mediated abscopal effects

**Fig. 2:**
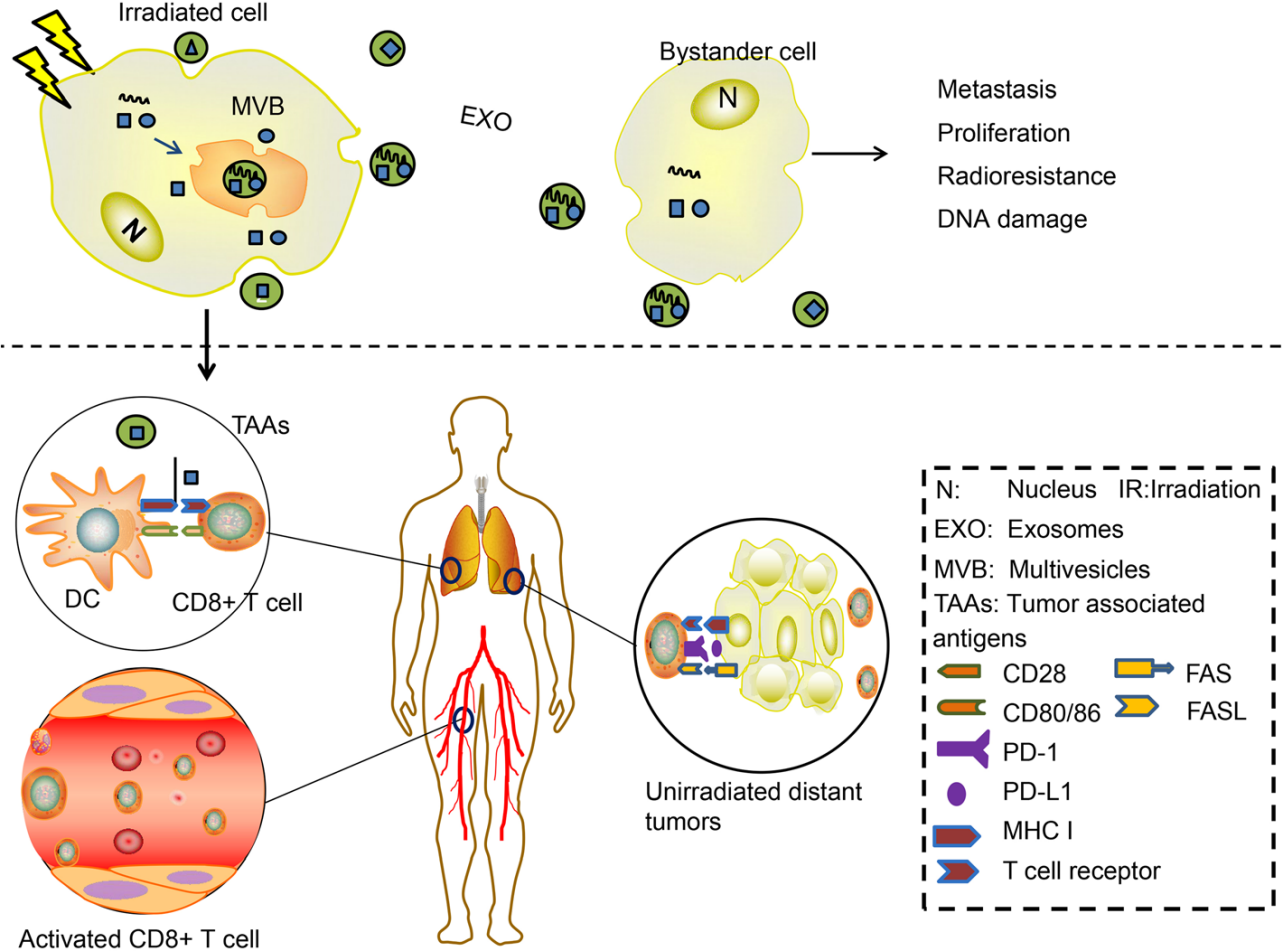
The role of exosomes in tumour immunity after IR. IR directly damages irradiated cells and alters their components. In response to IR, irradiated cells suffering immunogenic death (ICD) produce and release a set of cytokines and chemokines. IR-induced molecules can be sorted into exosomes directly or stochastically during exosome formation. After secretion into the tumour microenvironment (TME), exosomes can be taken up by unirradiated bystander cells. The uptake of exosomal contents, such as DNA, microRNA, and proteins, results in altering cell signalling pathways, including metastasis, proliferation, and radioresistance, and induces genetic damage in recipient cells. For example, after IR, exosomes transfer increased numbers of TAAs with increased diversity to DCs stimulating a robust tumour-specific immune response in which specific CD8+ T cells travel to recognize and attack both primary and distant metastatic tumours. This finding may explain the abscopal effect to some extent

The possibility of exosome-mediated abscopal effects has been examined in different tumours. Scientists have revealed the synergistic effect of exosomal contents, such as DNAs, RNAs, microRNAs, and proteins, in inducing the abscopal effect [102]. Abscopal effects can cause damage, mainly chromosomal or genomic instability in unirradiated cells. It is believed that factors that mediate this cellular communication can be transmitted by gap junctions or exosomes. IR induces several dysregulated proteins and nucleic acids within cells. During exosome formation, effectors, such as proteins, microRNAs, and DNAs, are packaged into these vesicles. IR-affected exosomes are then released to the TME and access adjacent cells through exosomal migration and internalization, thereby inducing abscopal effects on distant nontargeted cells [103–105] (Fig. 2).

Exosome components have different roles in the abscopal effect, such as DDR modulation and inflammation regulation [105]. Exosomes purified from irradiated cells contain specific RNAs to induce early and late chromosomal breaks in bystander cells. One study showed that radiating MCF7 cells with 2 Gy and then treating bystander cells with RNase or exosomes isolated from the radiated cells inhibited or increased the levels of genomic damage, respectively [106]. In another study, unirradiated MCF7 cells treated with exosomes from irradiated and bystander progeny cells exhibited significant levels of DNA damage. When exosomal RNA and proteins are inhibited, this effect can be eliminated. This study showed that exosomal RNA and protein are effective in initiating abscopal effects, which can be long-lived [92]. Consistent with studies that the abscopal effect was exosome-mediated, exosomes that released by irradiated breast cancer cells in a mouse model could transfer DNA strands to DCs and induce the upregulation of costimulatory molecules and IFN-γ activation in DCs, which could exert a systemic antitumour effect in a tumour model after IR [52]. Additionally, the significance of exosomal miRNAs in eliciting abscopal effects has been documented. miR-21 is a kind of miRNA that functions in abscopal effects [106]. In an MRC-5 cell model, marked upregulation of miR-21 in both irradiated cells and bystander cells was observed, and this study also confirmed that exosomes generated from irradiated MRC-5 cells transferred miRNA-21 to target cells, contributing to DNA damage and chromosome aberrations [91]. In another experiment, researchers first identified that miR-7-5p in exosomes collected from 2 Gy-irradiated human bronchial epithelial BEP2D cells could induce autophagy in recipient cells. This autophagy occurs mainly through miR-7-5p-mediated modulation of EGFR and downstream signalling pathways such as Akt-mTOR. This finding can explain why RT can damage adjacent normal tissues and may also damage tumour cells to some extent [107]. The radiation-induced abscopal effect is a damage response in unirradiated cells that occurs via intercellular communication through gap junctions or specific contents in exosomes that are produced by irradiated cells. These results support the involvement of specific exosomal contents in expanding the abscopal effect during RT (Table 1).

**Table 1:** Mechanisms of exosomes-mediated abscopal effects

Exosome cargo	Cell type	Functions	References
TAAs (CDCP1) DAMPs (Hsp70,Hsp90)	H22 hepatoma and 4 T1 breast cancer cells	Activate antigen-specific CD4 and CD8 T cells via cross-presentation pathways, enhance tumour infiltration of CD4 and CD8 T cells	[9]
Mart-1/MelanA tumour antigens, Tyrosinase-related protein, HSP70,	Melanoma cells	Transfer MHC-I–peptide complexes and/or whole antigens to DCs to promote CTL activation	[7, 47]
ANXA1, ANAX2, ITGB1, ITGA3, FN1, CTNNB1, APOH	MSCs	Activate leukocyte adhesion to tumour cells to limit tumour growth, induce tumour cells apoptosis and modulate radiotherapeutic efficacy.	[45]
dsDNA	BALB/C mouse derived mammary carcinoma cell	Activate IFN-I via cGAS/STING pathway in DCs	[52]
MiR-21	lung fibroblast MRC5 cell	Depress target gene (bcl-2) expression, increase chromosomal aberration and DNA damage in bystander cells	[91]
Proteins RNAs	breast epithelial cancer MCF7 cell breast epithelial cancer MCF7 cell	Cause inflammation and chromosomal damage in unirradiated cells Changes in epigenetics, delayed damage in unirradiated cells	[92]
MiR-7-5p	human bronchial epithelial BEP2D cell	Induce autophagy in non-targeted cells by EGFR/Akt/mTOR signalling pathway	[107]

*TAAs* Tumour associated antigens; *CDCP1* CUB Domain containing protein 1; *DAMPs* Damage associated molecular patterns; *Hsp70* Heat shock protein 70; *Hsp90* Heat shock protein 90; *ANXA1* Annexin A1; *ANXA2* Annexin A2; *ITGB1* Integrin subunit beta 1; *ITGA3* Integrin subunit alpha 3; *FN1* Fibronectin1; *CTNNB1* Catenin beta 1; *APOH* Apolipoprotein H; *MSCs* Mesenchymal stem cells

However, exosomes are also closely linked to radiation resistance. After analysing the rate of DNA double-strand break (DSB) repair in irradiated HNSCC, researchers demonstrated that exosomes from irradiated donor cells could promote cell survival by increasing DNA repair and increase radiation resistance [92]. These studies inspire us that target the exosomal regulation mechanisms associated with radiation, and exosome formation may be a new strategy of enhancing the effectiveness of RT and countering radioresistance.

## Conclusion

It is intriguing to exploit the biological functions of exosomes, as exosomes convey genomic instability and abscopal effects to inhibit tumour regression but are also associated with radioresistance via their contents, such as RNAs, microRNAs, and proteins. Different exosomal cargoes result in various effects. Research on exosomes in RT is new and hopeful. Radiation affects not only the formation but also the secretion of exosomes, thereby influencing the communication, signalling pathways or gene expression of recipient cells and making exosomes an ideal predictive biomarker of RT response monitoring. Radiation-affected exosomes can release different cargoes to recipient cells and exert different biological effects to modulate radiosensitivity. However, the impact on recipient cells can be synergistic or opposing to the effects of different exosomes due to their heterogeneity.

In short, exosomes are robust immune regulators of tumours during RT contributing to RT efficacy, and are crucial in tumour control. However, we still lack universal, precise, and suitable technologies to purify and isolate specific exosomes or target exosomes with specific contents. The intracellular transport of exosomal contents and the molecular and cellular mechanisms of the interactions of exosomes with other cells during radiation have yet to be fully elucidated. Therefore, additional studies need to be done to elicit the best effects of RT. Focusing on exosomes as the targets of tumour radiation may be a choice of new technologies. On the one hand, enhance the immunostimulatory effects of exosomes in RT on innate and adaptive immunity, inhibiting the suppressive molecules transferred by exosomes in the context of RT and the migration signalling pathways and ultimately avoiding radioresistance. On the other hand, since exosomes are endogenous, stable, and can be easily engineered or labelled, it is possible to use specific exosomes as targeted immunoregulators in cancer treatment. We predict that increasing the understanding of exosome contents and biology, along with standardized methods for exosome isolation, will facilitate improved understanding of exosome-mediated RT response mechanisms and enable the harnessing of exosomes as therapeutic targets. Verifying this hypothesis will ultimately contribute to the development of cancer treatment.

## Data Availability

Not applicable.

## Abbreviations

RT: Radiotherapy
TME: Tumour microenvironment
NK: Natural killer
Tregs: Regulatory T cells
MDSCs: Myeloid-derived suppressor cells
ICD: Immunogenic cell death
TAAs: Tumour associated antigens
APCs: Antigen presenting cells
DAMPs: Danger-associated molecular patterns
MHC I: Major histocompatibility complex class I molecules
CTL: Cytotoxic T cell
TCRs: T cell receptors
PD-1: Programmed cell death 1
DC: Dendritic cell
HMGB1: High mobility group box 1
IFN: Interferon
NKG2D: Natural-killer group 2 member D
IR: Irradiation
ICAM-1: Intercellular adhesion molecule-1
TNF: Tumour necrosis factor
TAMs: Tumour-associated macrophages
CTLA4: Cytotoxic T lymphocyte associated protein 4
TGF-β: Transforming growth factor-β
IL: Interleukin
HNSCC: Head and neck squamous cell carcinoma
MVBs: Multivesicular bodies
ILVs: Intraluminal vesicles
ESCRT: Endosomal sorting complex required for transport
TEXs: Tumour-derived exosomes
IEXs: Immune cell-derived exosomes
CEA: Carcino-embryonic antigen
HSP: Heat shock protein
CXCL-16: C-X-C motif chemokine ligand 16
CXCR6: C-X-C motif chemokine receptor 6
MSCs: Mesenchymal stem cells
MMP: Matrix metalloproteases
DEXs: Dendritic cell-derived exosomes
PD-L1: Programmed cell death ligand 1
CTGF: Connective tissue growth factor
IGFBP2: Insulin-like growth factor binding protein 2
DSB: Double-strand break
ICIs: Immune checkpoint inhibitors
